# Meta-analysis of randomized controlled trials on magnesium in addition to beta-blocker for prevention of postoperative atrial arrhythmias after coronary artery bypass grafting

**DOI:** 10.1186/1471-2261-13-5

**Published:** 2013-01-23

**Authors:** Xiaosan Wu, Congxia Wang, Jinyun Zhu, Chunyan Zhang, Yan Zhang, Yanhua Gao

**Affiliations:** 1 Department of Cardiovascular Medicine, the Second Affiliated Hospital of Medical School, Xi’an Jiaotong University, Xi’an, Shaanxi, 710004, PR China

**Keywords:** Magnesium, Beta-blocker, Postoperative atrial arrhythmia, Meta-analysis

## Abstract

**Background:**

Atrial arrhythmia (AA) is the most common complication after coronary artery bypass grafting (CABG). Only beta-blockers and amiodarone have been convincingly shown to decrease its incidence. The effectiveness of magnesium on this complication is still controversial. This meta-analysis was performed to evaluate the effect of magnesium as a sole or adjuvant agent in addition to beta-blocker on suppressing postoperative AA after CABG.

**Methods:**

We searched the PubMed, Medline, ISI Web of Knowledge, Cochrane library databases and online clinical trial database up to May 2012. We used random effects model when there was significant heterogeneity between trials and fixed effects model when heterogeneity was negligible.

**Results:**

Five randomized controlled trials were identified, enrolling a total of 1251 patients. The combination of magnesium and beta-blocker did not significantly decrease the incidence of postoperative AA after CABG versus beta-blocker alone (odds ratio (OR) 1.12, 95% confidence interval (CI) 0.86-1.47, *P* = 0.40). Magnesium in addition to beta-blocker did not significantly affect LOS (weighted mean difference −0.14 days of stay, 95% CI −0.58 to 0.29, *P* = 0.24) or the overall mortality (OR 0.59, 95% CI 0.08-4.56, *P* = 0.62). However the risk of postoperative adverse events was higher in the combination of magnesium and beta-blocker group than beta-blocker alone (OR 2.80, 95% CI 1.66-4.71, *P* = 0.0001).

**Conclusions:**

This meta-analysis offers the more definitive evidence against the prophylactic administration of intravenous magnesium for prevention of AA after CABG when beta-blockers are routinely administered, and shows an association with more adverse events in those people who received magnesium.

## Background

Atrial tachyarrhythmias are a common complication of cardiac surgery, with an incidence of 11% to 40% in patients after coronary artery bypass grafting (CABG) and more than 50% in patients after valvular surgery. Atrial fibrillation (AF), flutter, and multifocal atrial tachycardia are different forms of atrial tachyarrhythmias, which have the similar clinical implications in early recovery of patients after cardiac surgery [1]. Potential complications of postoperative atrial tachyarrhythmias (POAT) include thromboembolic events, hemodynamic compromise, delay of clinical recovery after cardiac surgery, and increased length of stay (LOS) and cost of hospitalization [2]. AF, occurring after CABG, is also a major determinant of postoperative stroke [3]. In addition to potentially increased risk of stroke and death, patients with recurrent or persistent atrial arrhythmia (AA) require additional medications, including systemic anticoagulation. Physicians and surgeons have been actively searching for effective strategies to reduce the incidence of postoperative AA. Several pharmacologic agents have been used to prevent postoperative supraventricular arrhythmias with varying degrees of success. Importantly, only beta-blockers and amiodarone have convincingly been shown to decrease its incidence [4,5]. Although moderate-dosage corticosteroid should be considered for the prevention of AF in high-risk patients undergoing cardiac surgery, the interaction between corticosteroids, beta-blockers, and amiodarone requires further study [6]. The preoperative administration of digoxin, calcium channel antagonists, and procainamide has been disappointing [4,7].

Hypomagnesemia has been observed after cardiac surgery [8,9], and most studies have shown that the administration of magnesium decreases the incidence of AA after cardiovascular surgery [10-13]. But the effectiveness of magnesium has been more controversial, because that evidence comes from multiple small, underpowered trials, with conflicting results, probably because of differences in study design, including the using of beta-blockers, although a recent meta-analysis concluded that magnesium is an effective prophylactic agent for prevention of postoperative AF [14].

To evaluate the effect of magnesium as a sole or adjuvant agent with currently used prophylactic drugs such as beta-blocker in suppressing postoperative AA, several randomized controlled trials (RCTs) have been examined. However, the results were controversial, which could possibly be attributable to lack of statistical power in individual studies. Therefore, we conducted a meta-analysis of RCTs to evaluate the effect of magnesium on the prevention of postoperative AA, the hospital LOS, mortality and adverse effects in addition to beta-blocker after CABG.

## Methods

### Publication search and inclusion criteria

This meta-analysis of RCTs was performed in accordance with the Quality of Reporting of Meta-analyses (QUOROM) consensus guidelines and according to a protocol that prespecified outcomes, search strategies, inclusion criteria, and statistical analyses [15]. We identified studies by a literature search of the PubMed, Medline, ISI Web of Knowledge, Cochrane library databases and online clinical trial database up to May 2012 with the following key words: “atrial arrhythmias”, “atrial tachyarrhythmias”, “atrial fibrillation” or “atrial flutter” plus “coronary artery bypass grafting” plus “magnesium” plus “beta-blocker”, “beta-blockade” or “β-blocker”. In addition, we reviewed the reference lists from all relevant articles to identify additional studies if necessary. All searches were conducted independently by 2 authors. The results were compared, and any questions or discrepancies were resolved through iteration and consensus.

Criteria for study inclusion: (i) the design was a prospective, RCT; (ii) both on-pump and off-pump CABG techniques were included and no limitations were applied to the number of the grafts used; (iii) all patients were in sinus rhythm at the time of surgery; (iv) the intervention was magnesium, as a bolus or continuous infusion, with a total dose beyond 35 mmol, given as a prophylactic measure (before the onset of AA) in the intervention group. The intervention group used magnesium in addition to beta-blocker, and the comparison group just used the same type of beta-blocker; and (v) the primary outcome was the incidence of AA after CABG (measured using a continuous electrocardiogram (ECG) monitoring). Studies that met any one of the following criteria were excluded: (i) unspecified methods of detection of AA or unspecified process of follow-up; (ii) other than magnesium and beta-blocker, the patients using any other anti-arrhythmic drugs (e.g. amiodarone) as prophylactic measures; and (iii) patients with a history of AA or ventricular arrhythmias or any rhythm other than sinus rhythm on the electrocardiogram obtained the evening before surgery.

### Data extraction and quality assessment

Trials selected for review were screened for information about patient characteristics, details of administration, process of treatment, efficacy in preventing postoperative AA, LOS, mortality and adverse effects. All data were extracted by one author and checked for accuracy by another author independently. Disagreements were resolved by consensus, and if necessary, a third author was required to assess it. We used the Jadad score [16] to assess the methodological quality of the included studies. The range of possible scores was 0 to 5.

### Statistical methods

One author entered related data into the meta-analysis software package (RevMan 5.1, the Cochran Collaboration, Information Management System, http://ims.cochrane.org/RevMan). The second author crosschecked the printout against his own data abstraction forms. All statistical analyses were performed using the RevMan5.1 package. Clinical homogeneity of included studies was considered first, by assessing study population, intervention group, comparison group and outcome. For dichotomous outcomes, results are expressed as risk ratio with 95% confidence interval (CI). For continuous outcomes, pooled data are described with the weighted mean difference (WMD) and 95% CI. Where significant heterogeneity was present, the studies were examined in detail for the reasons of heterogeneity. We assessed heterogeneity with *I*^*2*^, which describes the percentage of total variation across studies due to heterogeneity rather than chance. *I*^*2*^ can be calculated as: I^2^ = 100% × (Q-df)/Q(Q = Cochrane’s heterogeneity statistics, df = degrees of freedom). Negative values of *I*^*2*^ equaled zero, so that *I*^*2*^ ranged between 0% (ie, no observed heterogeneity) and 100%. High values would show increased heterogeneity [17]. Data were considered to be heterogeneous if the chi-square generated by RevMan heterogeneity test was associated with a *P* value <0.05. Where significant heterogeneity was present, attempts were made to explain the differences based on the patient clinical characteristics and interventions of the included studies. Random effects method was used to address heterogeneity where appropriate [18]. Where heterogeneity prohibited pooling, data were presented as a qualitative overview. For the outcome of interest (occurrence of AA after CABG), odds ratio (OR) with 95% CI was used. Fixed effects model was used for pooling where the trials were homogenous. Where heterogeneity was evident, random effects model was used for pooling [18].

## Results

### Characteristics of the included studies

**Figure 1:**
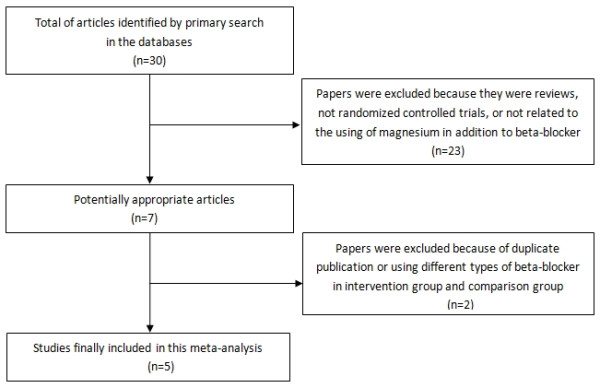
Summary of study selection and exclusion process.

Our initial data search yielded a total of 30 studies (Figure 1). We excluded 24 studies as they did not meet our inclusion criteria. Six potentially relevant studies were identified [19-24]. One study was excluded because of duplicate publication [24]. Thus, a total of 5 studies were included in this meta-analysis [19-23]. The publication dates spanned from 2000 to 2009. Patient enrolled in a single study ranged from 103 to 694 patients. The total number of randomized patients was 1251. The quality assessment and Jadad score evaluation of the included studies are listed in Table 1. The patient and study characteristics are shown in Tables 2 and 3, respectively. Most studies reported that magnesium in addition to beta-blocker had non-significant prevention of postoperative AA after CABG [19,21-23]. But one study showed that this strategy could significantly decrease the incidence of AF after CABG [20]. Three studies were conducted in USA [19,22,23], one in Italy [20], and the remaining one in Netherlands [21]. Regimens of magnesium and beta-blocker administrations varied and are summarized in Table 3. On hospital wards, detecting arrhythmia was performed by continuous ECG through postoperative day 2 to 5, and thereafter a daily ECG study were performed until hospital discharge for most trials.

**Table 1:** Quality features of 5 studies of RCTs assessed in the meta-analysis

**Study**	**Year**	**Design**	**Reporting of randomization**	**Generation of random sequence**	**Completeness of follow-up**	**Description of withdrawals**	**Quality score**
Bert et al. [19]	2001	NS	Y	Table of random numbers	Y	Y	4
Cook et al. [22]	2009	DB	Y	NA	Y	Y	4
Forlani et al. [20]	2002	NS	Y	computer-generated random code	Y	Y	4
Geertman et al. [21]	2004	DB	Y	NA	Y	Y	4
Solomon et al. [23]	2000	NS	Y	NA	Y	Y	3

DB: double-blind; NS: blinding characteristics of study not specified; NA: unable to assess; RCTs: randomized controlled trials; Y: yes.

**Table 2:** Patient characteristics (Mag + BB group/BB group) of randomized controlled studies

**Study**	**Number of patients**	**Mean age (years)**	**Men (%)**	**Mean LVEF (%)**	**Hypertens-ion (%)**	**Diabetes (%)**	**Preop BB (%)**	**Preop digoxin(%)**	**Preop CCB (%)**
Bert et al. [19]	69/71	62.3/63.8	82.6/76.1	48/49	NA	NA	75.4/76.1	21.2/21.2	17.2/18.5
Cook et al. [22]	347/347	64/64	85.6/83.6	NA	66.9/68	35.7/30.8	88.2/89	1.7/0.9	27.7/24.5
Forlani et al. [20]	52/51	62/64	91/82	55.3/54.7	63/72	25/38	36/45	7/3	14/16
Geertman et al. [21]	74/73	63.8/61.7	78.4/79.5	NA	32/30	13.5/8.2	90.5/86.3	NA	NA
Solomon et al. [23]	85/82	62/61	80/73	53/54	NA	NA	72/78	3.5/7.3	39/37

Mag: magnesium; BB: beta-blocker; CCB: calcium channel blocker; NA: data not available; LVEF: left ventricular ejection fraction; Preop: preoperative.

**Table 3:** Study characteristics

**Study**	**Infusion of Mag**	**Treatment initiation of Mag**	**Route of Mag**	**Treatment Duration of Mag (hours)**	**Total dose of Mag (mmol)**	**Type of BBs**	**Definitions of AA with continuous ECG**
Bert et al. [19]	Magnesium sulfate	Postop	Iv	96	49	Propranolol	POAT including AF, AFL, or a SVT that was sustained >5 minutes and warranted pharmacologic therapy
Cook et al. [22]	Magnesium sulfate	Preop	Iv	120	102	Atenolol	AA lasting >30 minutes or causing hemodynamic compromise requiring immediate intervention
Forlani et al. [20]	Magnesium sulfate	Preop	Iv	144	37	Sotalol	AF that required treatment for symptoms or hemodynamic deterioration
Geertman et al. [21]	Magnesium chloride	Preop	Iv	36	75	Sotalol	POAT including AF,AFL, and multifocal atrial tachycardia
Solomon et al. [23]	Magnesium sulfate	Postop	Iv	24	73	Propranolol	AF lasting longer than 1 hour or requiring therapy as a result of hemodynamic compromise

Mag: magnesium; BBs: beta-blockers; Iv: intravenous; Preop: preoperatively; Postop: postoperatively; AA: atrial arrhythmias; POAT: postoperative atrial tachyarrhythmias; AF: atrial fibrillation; AFL: atrial flutter; SVT: supraventricular tachycardia.

### The pooled treatment effect

**Figure 2:**
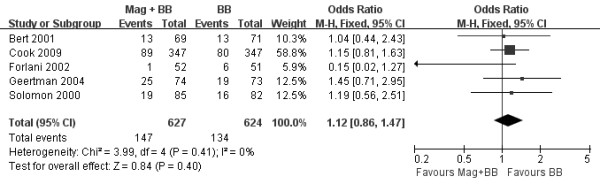
**Forest plot showing the incidence of postoperative AA after CABG.** Values are given as the odds ratio [95% confidence interval (CI)] as determined by the fixed effects model. Mag: magnesium; BB: beta-blocker.

**Figure 3:**
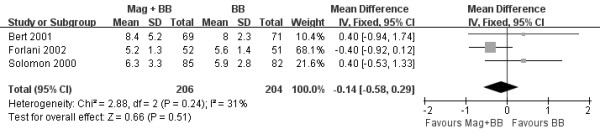
**Forest plot showing the effect on hospital LOS (days) after CABG.** Values are given as the mean difference [95% confidence interval (CI)] as determined by the fixed effects model. Mag: magnesium; BB: beta-blocker.

**Figure 4:**
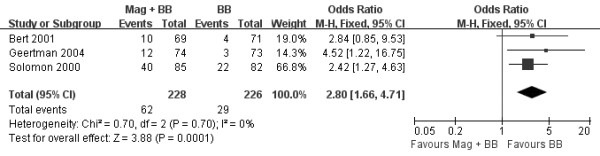
**Forest plot showing the adverse events following CABG.** Values are given as the odds ratio [95% confidence interval (CI)] as determined by the fixed effects model. Mag: magnesium; BB: beta-blocker.

The OR used for estimating the combination of magnesium and beta-blocker versus beta-blocker alone to prevent postoperative AA is shown in Figure 2. The pooled OR and risk ratio (RR) of all studies, by the fixed effects model, did not show that combination of magnesium and beta-blocker significantly decreased the incidence of postoperative AA after CABG verse beta-blocker alone (OR 1.12, 95% CI 0.86-1.47, *P* = 0.4; RR 1.09, 95% CI 0.89-1.34, *P* = 0.4) and little heterogeneity was indicated (*P* = 0.41, *I*^*2*^ =0% and *P* = 0.44, *I*^*2*^ =0%, respectively). Three trials reported the effect on hospital LOS with a total of 410 patients (Figure 3) [19,20,23]. LOS after CABG was similar between the two groups (WMD −0.14 days of stay, 95% CI −0.58 to 0.29, *P* = 0.51), although minor heterogeneity was indicated (*P* = 0.24, *I*^*2*^ =31%). Three trials reported data on postoperative mortality [19,21,23], with a total of 454 patients. Overall mortality was not significantly affected by magnesium in addition to beta-blocker (OR 0.59, 95% CI 0.08-4.56, *P* = 0.62). The risk of postoperative adverse events was higher in the combination of magnesium and beta-blocker group than beta-blocker alone group (OR 2.80, 95% CI 1.66-4.71, *P* = 0.0001; Figure 4) and little heterogeneity was indicated (*P* = 0.70, *I*^*2*^ =0%).

### Sensitivity/subgroup analysis

**Figure 5:**
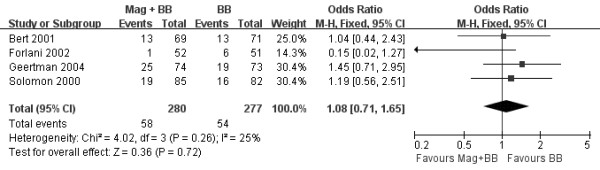
**Forest plot showing the incidence of POAT after CABG.** Values are given as the odds ratio [95% confidence interval (CI)] as determined by the fixed effects model. Mag: magnesium; BB: beta-blocker.

We used subgroup analysis to explore the effect of POAT, including AF in Figure 5[19-21,23]. The OR of these studies, by the fixed effects model, also did not show that combination of magnesium and beta-blocker significantly decreased the incidence of POAT after CABG versus beta-blocker alone (OR 1.08, 95% CI 0.71-1.65, *P* = 0.72). There was no statistically significant heterogeneity across the studies (*P* = 0.26, *I*^*2*^ =25%).

To assess possible differences related to the dose of magnesium administered, these trials were subdivided into low (<50 mmol) and high (> =50 mmol) dosages of magnesium. In the 2 trials with low dose magnesium (243 patients) [19,20], the percentage of patients with AA was increased from 11.6% in the combination of magnesium and beta-blocker group to 15.6% in the beta-blocker alone group, but the result was not significantly increased (OR 0.71, 95% CI 0.34-1.51, *P* = 0.38) (Table 4). In another 3 trials with large dose magnesium (1008 patients) [21-23], the percentage of patients with AA was not significantly decreased by using magnesium in addition to beta-blocker (OR 1.2, 95% CI 0.9-1.6, *P* = 0.21) (Table 4).

**Table 4:** Sensitivity and subgroup analysis

**Intervention**	**RCTs (n)**	**OR**	**95% CI**	***P*-value for heterogeneity**	***P*-value for overall effect**
Low dose of Mag	2	0.71	0.34-1.51	0.09	0.38
High dose of Mag	3	1.2	0.90-1.60	0.85	0.21
Sotalol	2	1.03	0.55-1.96	0.05	0.92
Other BBs	3	1.14	0.85-1.53	0.97	0.38

Mag: magnesium; BBs: beta-blockers; RCTs: randomized controlled trials; OR: odds ratio; CI: confidence interval.

Of note, sotalol, a new type of beta-blocker [25], is not only a beta-blocker but also a potent potassium channel blocker. So we conducted a subgroup analysis and subdivided trials into sotalol group and other beta-blockers group. In the 2 trials with sotalol (250 patients) [20,21], the percentage of patients with AA was decreased from 20.6% in the combination of magnesium and sotalol group to 20.2% in the sotalol alone group (OR 1.03, 95% CI 0.55-1.96, *P* = 0.92) (Table 4). In another 3 trials with other beta-blockers (1001 patients) [19,22,23], the percentage of patients with AA was decreased from 24.2% in the combination of magnesium and other beta-blockers group to 21.8% in other beta-blockers alone group (OR 1.14, 95% CI 0.85-1.53, *P* = 0.38) (Table 4).

## Discussion

AF is a common complication of CABG, especially in the elderly [26,27]. Although long-term sequelae of postoperative AF are unusual, it frequently results in an increased length and cost of hospitalization. Aranki and colleagues found that the length of hospitalization directly attributable to AF was 4.9 days [27]. This translated into more than $10,000 in hospital charges per patient [23]. Therefore, any intervention that would reduce the incidence of postoperative AF or AA would result in a tremendous economic benefit.

AA is a common complication of CABG and beta-blockers have been shown to decrease the incidence of postoperative AA. Although magnesium has proven effective in reducing ventricular tachyarrhythmias and early mortality in acute myocardial infarction [28], its role in suppressing POAT remains controversial. This meta-analysis showed that magnesium could not significantly decrease the incidence of postoperative AA after CABG in addition to beta-blocker. In order to diminish the extent of AA, we used a subgroup analysis to explore the influence of POAT, and showed the same result.

Although the meta-analysis by Miller et al. [14] concluded that magnesium administration is an effective prophylactic measure for the prevention of postoperative AF, only 4 of the 20 studies included in the analysis were clearly in favor of magnesium administration [12,20,29,30], with 7 studies showing no reduction in AF with magnesium prophylaxis [19,31-36]. There are 4 potential reasons for these different results, including: (i) the potential for β error secondary to small sample sizes; (ii) different definitions of AF; (iii) different doses of magnesium administered; and (iv) different use of concomitant beta-blocker [20]. In order to avoid the above shortness to some extent, the 5 RCTs in this meta-analysis have same characteristics, including: (i) the total dose of magnesium beyond 35 mmol; (ii) the intervention group and the comparison group used the same type of beta-blocker; and (iii) AA was detected by continuous ECG monitoring. As a result of the overwhelming data supporting the benefit of beta-blockers and some encouraging studies on the use of magnesium, we sought to determine whether magnesium as a sole or adjuvant agent in the prevention of AA after CABG. As a result, the combination of magnesium and beta-blocker did not significantly reduce the incidence of postoperative AA compared with beta-blocker alone.

One issue with the use of antiarrhythmic agents to prevent postoperative AA is that the majority of patients does not develop postoperative AA after cardiac surgery but would still be exposed to potential side effects of prophylactic intervention. From our meta-analysis, the risk of postoperative adverse events was higher in the combination of magnesium and beta-blocker group than beta-blocker alone group. The majority of adverse events were bradycardia and hypotension. An explanation for this phenomenon could be that intravenous administration of magnesium prolongs sinoatrial node conduction time, atrioventricular nodal refractory period, and PR and atrial-His intervals, as shown in electrophysiologic studies in healthy human subjects [37]. It is assumed that these effects are amplified when magnesium is combined with beta-blocker. Further specific studies are needed to evaluate this hypothesis.

It has been reported that sotalol is not only a beta-blocker but also a potent potassium channel blocker. Therefore, we conducted a subgroup analysis and subdivided trials into sotalol group and other beta-blockers group. Both sotalol group and other beta-blockers group showed the same result that magnesium in addition to beta-blocker could not significantly decrease the incidence of postoperative AA. At the same time, trials were subdivided into low and high dosages of magnesium, and showed the same result. Because there was no use of amiodarone in all the included trials, so we can exclude the impact of amiodarone on the results of this meta-analysis.

Since postoperative AA has been associated with LOS extended, greater risk of major morbidity and accompanying increases in hospital costs, we conducted subgroup analyses about LOS and mortality. From this meta-analysis, magnesium in addition to beta-blocker did not significantly reduce the LOS in this meta-analysis. Since only 3 studies provided data on LOS, additional studies or data are warranted. Three trials reported data on mortality, and the result showed that the overall mortality was not affected by magnesium administration in addition to beta-blocker.

### Limitations

This meta-analysis is limited by the lack of studies and complete availability of relevant data, particularly for LOS, mortality and adverse effects. Each included trial had different categories of adverse events. For example, the trial by Bert et al. [19] demonstrated postoperative adverse events that included myocardial infarction and ventricular ectopic activity, whereas the trial by Geertman et al. [21] only included serious bradyarrhythmias and nonsustained ventricular tachycardia. The research by Solomon et al. [23] showed the adverse events including bradycardia and hypotension. Due to the limitation in the number of included studies, we did not have subdivision of adverse events, such as bradycardia and hypotension which often occur in the studies. According to the Cochrane Handbook [38], tests for funnel plot asymmetry should be used only when there are at least 10 studies included in the meta-analysis, because when there are fewer studies the power of the tests is too low to distinguish chance from real asymmetry. Therefore, Because of 5 articles in this meta-analysis, the funnel plot was not done in this meta-analysis. In addition, the number of patients in most studies was small.

## Conclusions

Based on this meta-analysis of RCTs, we can conclude that magnesium administration isn’t significantly effective for reducing postoperative AA, hospital LOS and mortality. Moreover, we observed an association with more adverse events in those people who received magnesium and beta-blocker. The combination of magnesium and beta-blocker does not appear to offer incremental benefit over beta-blocker alone in the prevention of AA. Considering the higher adverse effects, the combination of magnesium and beta-blocker should be contraindicated in patients undergoing CABG. Beta-blockers or amiodarone should be used for the prevention of postoperative AA. Therefore, we believe that this meta-analysis offers the more definitive evidence against the prophylactic administration of intravenous magnesium for prevention of AA after CABG when beta-blockers are routinely administered. In addition, considering limitations of this meta-analysis, more RCTs are needed to analyze and do more subgroups analysis in order to get much more accurate data. Larger studies to further confirm these clinical benefits and evaluate their cost-effectiveness would be worthwhile.

## Competing interests

The authors declare that they have no competing interests.

## Authors’ contributions

XW conceived the study, participated in the design, collected the data, and drafted the manuscript. CW conceived the study, participated in the design, and helped to draft the manuscript. JZ collected the data, and drafted the manuscript. CZ and YZ collected the data, and performed statistical analyses. YG helped to draft the manuscript. All authors read and approved the final manuscript.

## Pre-publication history

The pre-publication history for this paper can be accessed here:

http://www.biomedcentral.com/1471-2261/13/5/prepub
